# Ferric Reducing Antioxidant Power and Square Wave Voltammetry for Assay of Low Molecular Weight Antioxidants in Blood Plasma: Performance and Comparison of Methods

**DOI:** 10.3390/s91109094

**Published:** 2009-11-17

**Authors:** Miroslav Pohanka, Hana Bandouchova, Jakub Sobotka, Jana Sedlackova, Ivana Soukupova, Jiri Pikula

**Affiliations:** 1 Centre of Advanced Studies and Department of Toxicology, Faculty of Military Health Sciences, University of Defence / Trebesska 1575, 50001 Hradec Kralove, Czech Republic; 2 Department of Veterinary Ecology and Environmental Protection, Faculty of Veterinary Hygiene and Ecology, University of Veterinary and Pharmaceutical Sciences Brno, Palackeho 1/3, 612 42 Brno, Czech Republic; E-Mails: bandouchovah@vfu.cz (H.B.); sedlackovaj@vfu.cz (J.S.); soukupovai@vfu.cz (I.S.); pikulaj@vfu.cz (J.P.); 3 Department of Biological and Biochemical Sciences, Faculty of Chemical Technology, University of Pardubice, Studentska 95, 532 10 Pardubice, Czech Republic; E-Mail: sobotka.jakub@seznam.cz (J.S.)

**Keywords:** lead intoxication, Cinereous vultures, glutathione, ascorbate, uric acid, analytical methods

## Abstract

The purpose of the present study was to employ two methods—square wave voltammetry (SWV) performed on screen printed sensors and ferric reducing antioxidant power (FRAP)—as suitable tools for the assay of low-molecular-weight antioxidants (LMWAs). LMWAs were assayed by both methods and the resulting data were statistically compared. Plasma samples from five Cinereous vultures accidentally intoxicated with lead were used to represent real biological matrices with different levels of LMWAs. Blood was collected from the birds prior to and one month after treatment with Ca-EDTA. SWV resulted in two peaks. The first peak, with the potential value of 466 ± 15 mV, was recognized as ascorbic and uric acids, while the second one (743 ± 30 mV) represented glutathione, tocopherol, ascorbic acid and in a minor effect by uric acid, too. Contribution of individual antioxidants was recognized by separate assays of LMWA standards. Correlation between peaks 1 and 2 as well as the sum of the two peaks and FRAP was analysed. While peak 1 and the sum of peaks were in close correlation to FRAP results (correlation coefficient of 0.97), the relation between peak 2 and FRAP may be expressed using a correlation coefficient of 0.64. The determination of thiols by the Ellman assay confirmed the accuracy of SWV. Levels of glutathione and other similar structures were stable in the chosen model and it may be concluded that SWV is appropriate for assay of LMWAs in plasma samples. The methods employed in the study were advantageous in minimal sample volume consumption and fast acquisition of results.

## 1. Introduction

Low-molecular-weight antioxidants (LMWAs) are a wide group of quite small molecules providing electrons to oxidizing agents which protect other molecules from oxidation in this way. LMWAs are oxidized during this process. The localization of various LMWAs is different. Glutathione is predominantly found in plasma, ascorbate in whole blood and alpha-tocopherol protects membranes [1]. Apart from LMWAs of non-enzymatic nature, the endogenous antioxidant defences also include enzymatic antioxidants critical for the control of reactive-molecular-species-mediated oxidative damage of biomolecules. However, the effect of enzymatic antioxidants is completely different to that of LMWAs. They cleave reactive oxygen species. Enzymes such as superoxide dismutase (SOD), catalase (CAT), glutathione peroxidase (GPx) and glutathione reductase (GR) may be mentioned as typical examples of enzymatic antioxidants [2,3]. It is common for the enzymatic antioxidants be assayed using standard biochemical procedures [4]. On the other hand, LMWAs are assayed using different analytical methods for functional groups in the antioxidant molecule and/or its redox power.

Some LMWA assays are based on the enzymatic cleavage of antioxidants. This approach was used, for example, by Vermeir *et al.* [5], who fabricated a calorimetric biosensor with trapped ascorbate oxidase and the biosensor was successfully used for the assay of the ascorbate in complex matrices such as food and drugs. Thiol-group containing antioxidants may be simply assayed by reaction with Ellman's reagent (5,5′-dithio-bis(2-nitrobenzoic acid)) which affords yellow coloured 5-thio-2-nitro-benzoic acid with an absorption at 412 nm [6,7]. Oxidized glutathione (GSSG) may be converted into the reduced form glutathione (GSH) using the enzyme GR and in this way it is possible to assay both forms [8].

The typical methods suitable for assay of the antioxidant potency are based on the reaction between antioxidants and chromogens which result in some colour change due to the redox reaction. Antioxidants may be assayed by the oxygen radical absorbance capacity (ORAC) based on fluorescein or beta-phycoerythrin. ORAC is suitable for estimation of the antioxidant content in foods and beverages [9]. The antioxidant power is counted in “Trolox equivalents”; the antioxidant power appears as a potency to prevent induced damage of fluorescein [10]. The ferric reducing antioxidant power assay (FRAP) is another method of wide suitability for assay of antioxidants *in vitro* as well as in organisms [11]. In some literature the FRAP method is referred to as the ferric reducing ability of plasma [12]. This assay is based on the reduction of Fe^III+^ to Fe^II+^ due to the action of antioxidants present. Subsequently, the Fe^II+^ formed may interact with 2,4,6-tris(2pyridyl)-*s*-triazine (TPTZ) providing a strong absorbance at 593 nm [13].

Many studies have confirmed the suitability of voltammetry to estimate antioxidant power [14]. Voltammetric assays are based on cyclic voltammetry (CV), square wave voltammetry (SWV), and differential pulse voltammetry (DPV). Antioxidants are oxidized under a typical voltage which results in a faradaic current proportional to the concentration of antioxidants [15]. Though the voltammetric assays are not as common as the photometric ones, we can demonstrate the suitability of voltammetric methods using some examples. Cyclic voltammetry was performed for assay of blood of animals exposed to sulphur mustard [16], patients exposed to the drug silymarine [17] and infected animals [18,19]. Adam *et al.* used SWV for the assay of flavonoids in biological matrices [20]. For example, DPV was performed for assay of lead and thiol rich proteins [21]. The suitability of voltammetry for assay of antioxidants in biological matrices was already extensively reviewed [22-24].

The present study was aimed at performing a comparison of two methods considered as suitable for assay of antioxidants in biological matrices, *i.e.*, FRAP and SWV. Real plasma samples were used as a model matrix and both methods were carried out in order to compare the data and evaluate the respective benefits.

## 2. Results and Discussion

First, commercially available LMWA standards were assayed in order to estimate their impact on the measured current. Trolox^®^, reduced glutathione, ascorbic acid and uric acid (Sigma-Aldrich, Prague Branch, Czech Republic) were assayed as the standard physiological antioxidants expected to be present in blood and plasma samples. The position of peaks was investigated in order to obtain standard values of redox peaks on the used screen printed electrodes and 1 mM antioxidants solutions in phosphate buffered saline (PBS). The observed peak positions are listed in Table 1. The standard LMWAs provided peaks at different positions. The peaks were divided into two groups: A and B. The potential range was divided for better orientation: A ≤ 600 mV and B > 600 mV. Two antioxidants appeared bivalent in the SWV assay, *i.e.*, ascorbic and uric acids. Though the ascorbic acid was oxidised at the lowest potential of the tested antioxidants: 310 mV, the second peak with a similar height of 699 mV was achieved. Contrary to this, uric acid was primarily oxidized at 452 mV. The second peak at 770 mV was nearly ten times lower when compared with the first one. Glutathione and trolox were oxidized at potentials of 764 and 717 mV, no other peak was found for these two antioxidants.

The simultaneous assay of a mixture containing equimolar substitution of glutathione, Trolox^®^ and uric acid (each 333 μM) was carried out in order to investigate any additive effects on the observed peaks. Two peaks were found. The first peak was found at 471 ± 23 mV and the second one at 765 ± 35 mV. SWV was thus found reliable not only when assaying individual LMWAs but also their mixture. Real samples were assayed in the next part of the study.

Plasma samples collected from five Cinereous vultures (*Aegypius monachus*) accidentally exposed to lead and treated by Ca-EDTA were used in the present study in order to simulate real conditions. Exposure of the vultures resulted in different blood lead levels. The samples were assayed using the FRAP and SWV methods. The FRAP method is considered a routinely available procedure suitable for the evaluation of antioxidants. Regression analysis between values provided by the FRAP and SWV methods was performed. LMWAs can counteract lead intoxication [22] and, on the other hand, depletion of LMWAs may enhance the impact of toxic metals [26]. Levels of LMWAs varied in the organisms' response to lead exposure. However, as only analytical and comparison issues were the objective of the present study, and the health effects of lead exposure in vultures will be presented elsewhere.

**Figure 1.**
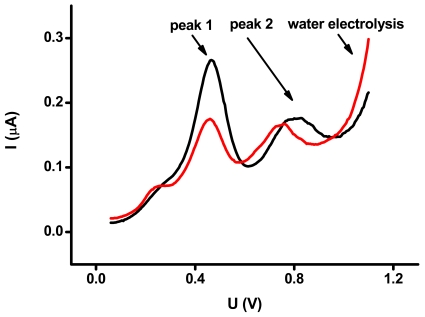
Voltammograms of vultures' plasma samples. Peak 1 (466 ± 15 mV), peak 2 (743 ± 30 mV) as well as water electrolysis region are indicated by arrows. The voltammogram depicted by black line was achieved by assay of the plasma sample with the FRAP value of 1,850 μmol/L, the red line indicates the plasma samle with the FRAP level of 1,350 μmol/L.

The FRAP assay of the vultures' plasma samples resulted in a range of values from 651 to 1,846 μmol/L. Apart from the highest values, the lower levels correspond with those found in healthy humans [27]. Plasma samples were also assayed by the SWV method and the typical square wave voltammograms with two peaks are shown in Figure 1. The first and second peaks were at 466 ± 15 and 743 ± 30 mV, respectively. The LMWAs appear in voltammograms as a typical wave in the anodic range. According to various investigators, the peak at the lower potential is due to uric and ascorbic acids [14], while thiol-containing molecules such as glutathione are represented by the second peak [15]. These facts were confirmed by the above-mentioned experiments. The composition of the second peak can be wider, as the participation of uric acid, tocopherol (in optimisation represented by water soluble Trolox^®^), glutathione and ascorbic acid on the second peak formation could be expected. Water electrolysis was found at the potential approximately 850 mV and higher. The upper potential 1 V was used just for excessive water electrolysis above this value. The lower potential was not, however, applied due to better focusing on peak 2.

**Figure 2.**
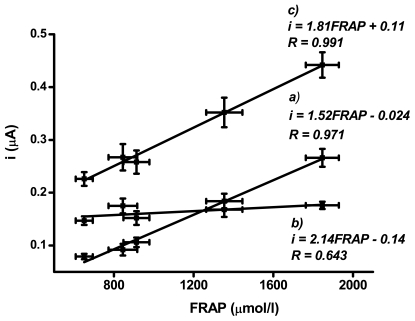
Comparison of FRAP and SWV from five vultures. Figures (a) and (b) represent the relation between peak 1 (466 ± 15 mV) and peak 2 (743 ± 30 mV) and FRAP values, respectively. Figure (c) represents a regression between the sum of peaks 1 and 2 and FRAP values. Error bars indicate standard error of mean for five time repeated measurement.

All plasma samples were assayed both by he FRAP and SWV methods and the obtained data were subjected to correlation analysis. The Origin 8 Software (OriginLab Corporation; Northampton, MA, USA) was used for data processing and calculation of correlation coefficients. Since two peaks were found when performing SWV, separate correlation analysis was done for peaks 1 and 2. Finally, the relation between the sum of peaks 1 and 2 and the FRAP data was analysed. Since the FRAP estimates all LMWAs and SWV can distinguish individual groups of antioxidants, the present effort to compare it uses the sum of peak heights. This way is suitable to estimate the contribution of individual antioxidants to the detected current. All correlations are presented in Figure 2.

Regression and correlation analyses of data obtained by the standard FRAP method and the SWV assay clearly demonstrates the suitability of SWV for the evaluation of LMWAs. These results could be expected when the date in [28] are considered. Zielinska *et al.* successfully compared Trolox^®^ equivalent antioxidant capacity with CV. In the present study a close correlation was found between peak 1 of the SWV method and the FRAP data, thus enabling extrapolation of either set of values using the regression, cf. graph (a) in Figure 2. The correlation coefficient was 0.971. This is a very good result when considering the fact that antioxidants were assayed in a complex biological matrix. When analysing the relation between peak 2 of the SWV method and the FRAP data [graph (b) in Figure 2], however, the correlation coefficient was only 0.643. The background of this lower correlation is probably due to the lower fluctuation of tocopherol, GSH and thiol-containing proteins in vultures and its lower contribution to the final antioxidant capacity of plasma. In order to confirm this fact, the modified Ellman's assay [4,29] using 5,5′-dithio-bis(2-nitrobenzoic) acid (DTNB) was used. Molar concentration of thiols was calculated from absorbance at 412 nm where the accumulated 5-thio-2-nitrobenzoic acid (TNB) absorbs light. Good correlation (R = 0.95) was found when peak 2 was plotted against concentration of TNB formed. This confirms the fact that the components of peak 2 may be identified as glutathione, cysteine and cysteine-rich proteins. On the other hand, as shown in graph (b), changes in glutathione were only minor. It seems that adaptive changes of GSH do not play an important role in the plasma of lead-intoxicated vultures after the Ca-EDTA treatment. Nevertheless, the evaluation of toxic effects of lead was not the main objective of the present study and this study did not determine LMWA levels shortly after exposure of the birds to lead. The last regression and correlation analysis was performed between the sum of peaks 1 and 2 and the FRAP data, cf. graph (c) in Figure 2. The result was similar to the correlation between peak 1 and the FRAP data, but even a closer correlation coefficient of 0.991 was found in this case. It may be stated that the most probable scenario of the LMWAs increase during exposure to lead is represented by the uric acid, in particular. This fact is supported by the exact position of peak 1 responding to the redox potential of the uric acid. The second fact supporting this idea is a minor influence of the uric acid on peaks above 600 mV.

Regarding the achieved results, validity and suitability of the SWV assay for the evaluation of LMWAs in complex biological matrices may be confirmed. The comparison of the SWV and FRAP methods confirmed not only good performance of SWV on screen printed sensors but also its superiority to FRAP due to the ability to distinguish between two essential types of LMWAs. The other undisputed advantage of SWV is the attainable rate of the assay. One SWV sample measurement takes about two minutes depending on the scan rate. There is no need to do any manipulation with the plasma sample such as incubation or addition of reagents. Moreover, the overall consumption of plasma is only 20 μL which is very favourable when compared to standard photometric methods [30]. It also appoint at electrochemical sensors as a convenient way suitable to analyzing of complex matrices [31,32]. It should also be mentioned that the study was carried out with disposable screen printed sensors enabling simple manipulation and a fast as well as reliable assay.

## 3. Experimental Section

### 3.1. Biological Samples

A total of five Cinereous vultures (*Aegypius monachus*) were exposed to dietary lead contamination in their aviaries. This accidental exposure resulted in lead toxicosis in the captive birds. Apart from standard toxicological tests, it was decided to use advanced laboratory tests including estimation of LMWAs levels and the FRAP assay to evaluate adverse effects of lead exposure on the birds. Ca-EDTA was administered to the birds as a suitable drug for lead intoxication treatment. For the purpose of this study, blood was collected from the ulnaris vein prior to and one month after the therapy with Ca-EDTA. The study is thus based on 10 samples collected from five birds. The age and body weight of the intoxicated vultures ranged from 8 to 36 years and 7 to 11.5 kg, respectively. Non-lead exposed birds (*i.e.*, four captive Cinereous vultures) were used as controls. Prior to therapy the lead mean content was 0.535 μg/g and it decreased to 0.236 μg/g a month after Ca-EDTA administration.

### 3.2. Ferric Reducing Antioxidant Power Assay

The FRAP assay was performed according to the references [13,33] with minor modifications. In the first round, the FRAP reagent was prepared as a mixture of 2.5 mL of 10 mM 2,4,6-tris(2pyridyl)-*s*-triazine (TPTZ) in 40 mM HCl and 2.5 mL of 20 mM FeCl_3_ in 25 mL of 0.1 M acetate buffer pH 3.6. The freshly prepared FRAP reagent was incubated at 37 °C for 10 minutes. 30 μL of a plasma sample was mixed with 200 μL of the FRAP reagent and added with distilled water up to 1 mL. After ten minutes of incubation, the mixture was centrifugated at 10,000 × *g*. A blank sample was prepared in the same way as described above but saline solution was used instead of the plasma sample. Absorbance of the supernatant was measured at 593 nm against blank.

### 3.3. Square Wave Voltammetry

Square wave voltammetry (SWV) was performed for assay of LMWAs in plasma. Screen printed sensors (BVT, Brno, Czech Republic) with platinum working (circle shaped, diameter 1 mm), silver covered with silver chloride reference electrode and platinum auxiliary on a ceramic support were used throughout the experiments. Measurements were processed by an electrochemical analyser (EmStat PalmSens, Houten, The Netherlands). Each electrochemical strip was used only once in order to avoid hysteretic interference. The electrochemical strip was fixed horizontally and electrodes were washed by ethanol prior to the assay. After that, electrodes were covered with 20 μL of the plasma sample. The potential was applied in a range from 0 to 1 V with potential step as well as potential amplitude 10 mV and frequency 1 Hz.

## 4. Conclusions

SWV was performed for the assay of LMWAs in plasma and compared with the corresponding FRAP data. A close correlation was found for both peaks observed by SWV versus the FRAP data. It is clear that SWV may be performed for the estimation of LMWAs in plasma with a similar sensitivity like the FRAP method. The undisputed advantage of SWV is the ability to distinguish two basic types of LMWAs, *i.e.*, ascorbic and uric acids and thiol-containing molecules. The FRAP procedure as well as some other photometric assays are not able to distinguish them and further tests are then necessary to characterize LMWAs in greater detail. SWV is very fast and no sample modification is required. It is advantageous for laboratories that are not assaying LMWAs routinely or examine low numbers of samples. In this study, screen printed sensors were used as a tool suitable for SWV. It was found that the here-performed sensors are applicable for assaying minimum amounts of samples within a short time.

## Figures and Tables

**Table 1.** Peaks observed by SWV for antioxidant standards of ascorbic acid, glutathione, trolox and uric acid.

**Antioxidant**	**Peak A (mV)**	**Peak B (mV)**	**Ratio of peak heights A:B**
ascorbic acid	310±55	699±34	1:1
glutathione	/	764±41	/
Trolox^®^	/	717±47	/
uric acid	452±36	770±25	10:1
